# Asking ‘why?’ enhances theory of mind when evaluating harm but not purity violations

**DOI:** 10.1093/scan/nsz048

**Published:** 2019-07-03

**Authors:** James A Dungan, Liane Young

**Affiliations:** 1 Booth School of Business, University of Chicago, Chicago, IL, 60637, USA; 2 Department of Psychology, Boston College, Chestnut Hill, MA, 02467, USA

**Keywords:** purity, theory of mind, construal, action identification, moral judgment

## Abstract

Recent work in psychology and neuroscience has revealed important differences in the cognitive processes underlying judgments of harm and purity violations. In particular, research has demonstrated that whether a violation was committed intentionally *vs* accidentally has a larger impact on moral judgments of harm violations (e.g. assault) than purity violations (e.g. incest). Here, we manipulate the instructions provided to participants for a moral judgment task to further probe the boundary conditions of this intent effect. Specifically, we instructed participants undergoing functional magnetic resonance imaging to attend to either a violator’s mental states (why they acted that way) or their low-level behavior (how they acted) before delivering moral judgments. Results revealed that task instructions enhanced rather than diminished differences between how harm and purity violations are processed in brain regions for mental state reasoning or theory of mind. In particular, activity in the right temporoparietal junction increased when participants were instructed to attend to why *vs* how a violator acted to a greater extent for harm than for purity violations. This result constrains the potential accounts of why intentions matter less for purity violations compared to harm violations and provide further insight into the differences between distinct moral norms.

## Introduction

Judgments of harm violations depend crucially on the mental states of the violator: harms caused intentionally are deemed more immoral than harms caused by accident. This difference is codified in US law where manslaughter carries a maximum sentence of 8 years in prison, whereas murder can result in a lifetime in prison or even a death sentence. It may seem surprising then that intentions appear to matter much less for judgments of other kinds of immoral behaviors; specifically, purity violations do not necessarily cause direct harm to others but are nevertheless condemned for being disgusting or unnatural and also, importantly, immoral (Inbar *et al.*, 2009; Rottman *et al.*, 2014). Research has consistently shown that the difference between moral judgments of intentional and accidental violations is greater for harm violations (e.g. murder) relative to purity violations (e.g. incest; Chakroff *et al.*, 2013; Dungan *et al.*, 2017; Young & Saxe, 2011). Strikingly, this intent effect holds even across eight small-scale societies tested in recent cross-cultural work (Barrett *et al.*, 2016). Here, we capitalize on previous work in social neuroscience to probe this intent effect further, ruling out potential reasons for why intent matters more for harm than purity.

Convergent evidence from social psychology and neuroscience has revealed important differences in the cognitive processes underlying judgments of harm and purity violations. Individual differences in how much people endorse harm and purity norms are associated with volumetric differences across the brain (Lewis *et al*., 2012), and violations of harm and purity norms elicit activity in distinct brain regions (Schaich Borg *et al*., 2008; Moll *et al*., 2005; Parkinson *et al*., 2011). In terms of behavior, judgments of purity violations (compared to judgments of harm violations) are relatively insensitive to potentially mitigating circumstances (e.g. the perpetrator was forced to commit the violation; Chakroff & Young, 2015; Piazza *et al*., 2013; Russell & Giner-Sorolla, 2011a,b). Additionally, as discussed above, whether an action was committed intentionally or accidentally matters relatively less for moral judgments of purity violations compared to harm violations (Barrett *et al*., 2016; Chakroff *et al*., 2013; Dungan *et al*., 2017; Young & Saxe, 2011).

Recent work has shed some light on the nature of the intent effect across the harm and purity domains. Our own functional magnetic resonance imaging (fMRI) work suggests that the intent effect does not seem to reflect some *post hoc* motivation to disregard intentions in order to deliver especially harsh judgments of purity violators, perhaps on the grounds of perceived character flaws (see Chakroff & Young, 2015). Using fMRI, we probed activity in the theory of mind (ToM) network (a set of brain regions involved in processing mental state information such as beliefs and intentions; Fletcher *et al*., 1995; Saxe and Kanwisher, 2003; Gobbini *et al*., 2007) as participants evaluated harm and purity violations in the scanner. Purity violations elicited less activity in the right temporoparietal junction (RTPJ), a key region for ToM, compared to harm violations (Chakroff *et al*., 2016). Critically, this difference in neural activity occurred even before participants knew whether an act was intentional or accidental, suggesting that people do not simply decide to assign less weight to the intentions of purity violators. Instead, at least part of the domain difference may be in the spontaneous recruitment of ToM for moral judgments.

While this prior work reveals that mental states, including intentions, matter less for moral judgments of purity *vs* harm violations, an open question is why this difference exists. One account is that the intent effect could reflect a difference in how people spontaneously approach judging harm and purity violations. Purity violations can elicit disgust (Haidt, Koller, & Dias, 1993; Russell & Giner-Sorolla, 2013), and people may want to avoid processing more complex details beyond the action itself, such as the violator’s intent. This account suggests that people prefer not to process the mental states of purity violators, but could do so if prompted. A second possible account for the intent effect is that judgments of purity violations are less sensitive than judgments of harm violations to information about mental states. This account suggests a relative lack of flexibility in judgments of purity *vs* harm violations in that even when mental state information is made salient, this information influences judgments of purity violations to a lesser degree compared to judgments of harm violations.

Here, we test these different accounts by manipulating the instructions provided to participants for a moral judgment task. Different task instructions have been shown to modulate activity in brain regions for ToM and social cognition more broadly (Kestemont *et al*., 2012; Ma *et al*., 2011; Van Overwalle, 2009). Of particular interest, instructing participants to attend to why an actor is behaving is associated with robust recruitment of ToM brain regions, compared to instructing participants to attend to how an actor is behaving, that is, to focus on the low-level mechanics of an action (Spunt & Adolphs, 2014; Spunt, Falk, & Lieberman, 2010; Spunt, Kemmerer, & Adolphs, 2015; Spunt, Satpute, & Lieberman, 2011). By focusing participants on either an actor’s mental states (why they did something) or their physical actions (how they did something), this task provides a clear way to directly probe the intent effect for judgments of harm and purity violations.

We offer a few key predictions for the possible impact of the how/why task instruction on the processing of harm and purity violations. These predictions apply primarily to activity in RTPJ, given prior work showing that RTPJ has the most selective response to information about an agent’s beliefs *vs* other socially relevant information about agents (e.g. Saxe & Kanwisher, 2003; Saxe & Powell, 2006; Saxe & Wexler, 2005) and is recruited to a greater extent when evaluating harm *vs* purity violations (Chakroff *et al*., 2016). First, we might observe similar recruitment of brain regions for ToM (particularly RTPJ) for both harm and purity violations when participants are explicitly instructed to attend to the violator’s mental states. Such a result would suggest that a difference in spontaneous mental state reasoning explains the intent effect across harm and purity, and that this difference is eliminated when participants are asked to attend to the mental states. Second, purity violations may continue to elicit less activity in brain regions for ToM than harm violations (as previously observed in RTPJ; Chakroff *et al*., 2016) even when participants are instructed to attend to the violator’s mental states. This result would support a critical difference in the way purity and harm violations are processed that is robust to attentional effects due to explicit instruction. Specifically, it would suggest that judgments of purity violations are indeed less sensitive to information about the violator’s mental states than judgments of harm violations.

## Methods

### Participants and procedures

Participants were 29 right-handed adults (*M*_age_ = 23.66, s.d._age_ = 4.39; 15 were female) recruited from the Greater Boston Area. All participants were native English speakers, had normal or corrected-to-normal vision and gave written informed consent in accordance with the Boston College Institutional Review Board. Additionally, participants reported no psychiatric disorders or history of learning disabilities. One participant was excluded due to scanner error and another for excessive movement (>8 mm). A final participant’s data set was excluded because her signal in RTPJ (the only ToM brain region identified in this participant by our functional localizer, see below) was highly erratic, changing by more than 5% from one time point to the next and was on average greater than 3 s.d.s from the average activity of all other participants. Thus, data from 26 participants were included in analyses.

Participants were scanned on a 3 T Siemens Tim Trio fMRI scanner (at the Martinos Imaging Center at MIT, Cambridge, MA) using thirty-six 3 × 3 × 3 mm near-axial slices (0.54 mm gap) covering the whole brain. Standard gradient echo planar imaging (EPI) procedures were used [time repetition (TR), 2 s; time echo (TE), 30 ms; flip angle (FA), 90°; field of view (FOV), 216 × 216; interleaved acquisition]. Anatomical data were collected with T1-weighted multiecho magnetization prepared rapid acquisition gradient-echo image sequences (TR, 2530 ms; TE, 1.64 ms; FA, 7^°^; 1 mm isotropic voxels; 0.5 mm gap between slices; FOV, 256 × 256).

Participants read a series of scenarios about a named protagonist engaging in some action. There were 70 distinct scenarios (see Supplementary Material): 28 depicting harm violations, 28 depicting purity violations and 14 depicting neutral actions. Harm violations included both physical harms (e.g. poisoning someone) and psychological harms (e.g. humiliating someone). Purity violations included both sexual violations (e.g. sex with a blood relative) and pathogen violations (e.g. eating maggots). Half of all scenarios depicted violations that the perpetrator committed intentionally, while the other half depicted violations that the perpetrator committed accidentally. Participants never saw both intentional and accidental versions of the same scenario.

Participants were given two distinct task instructions (the why task and the how task, adapted from Spunt, Falk, & Lieberman, 2010), counterbalanced across experimental runs and participants. For the why task, participants were instructed to think about why protagonists were doing what they were doing. While participants read the scenarios, they were instructed to think about one or more plausible motives for performing the action. For the how task, participants were instructed to think about how protagonists were behaving, that is, to think about one or more necessary parts of performing the action. Participants saw each scenario twice: once for the why task and once for the how task, meaning that each participant completed a total of 140 trials.

**Fig. 1:**
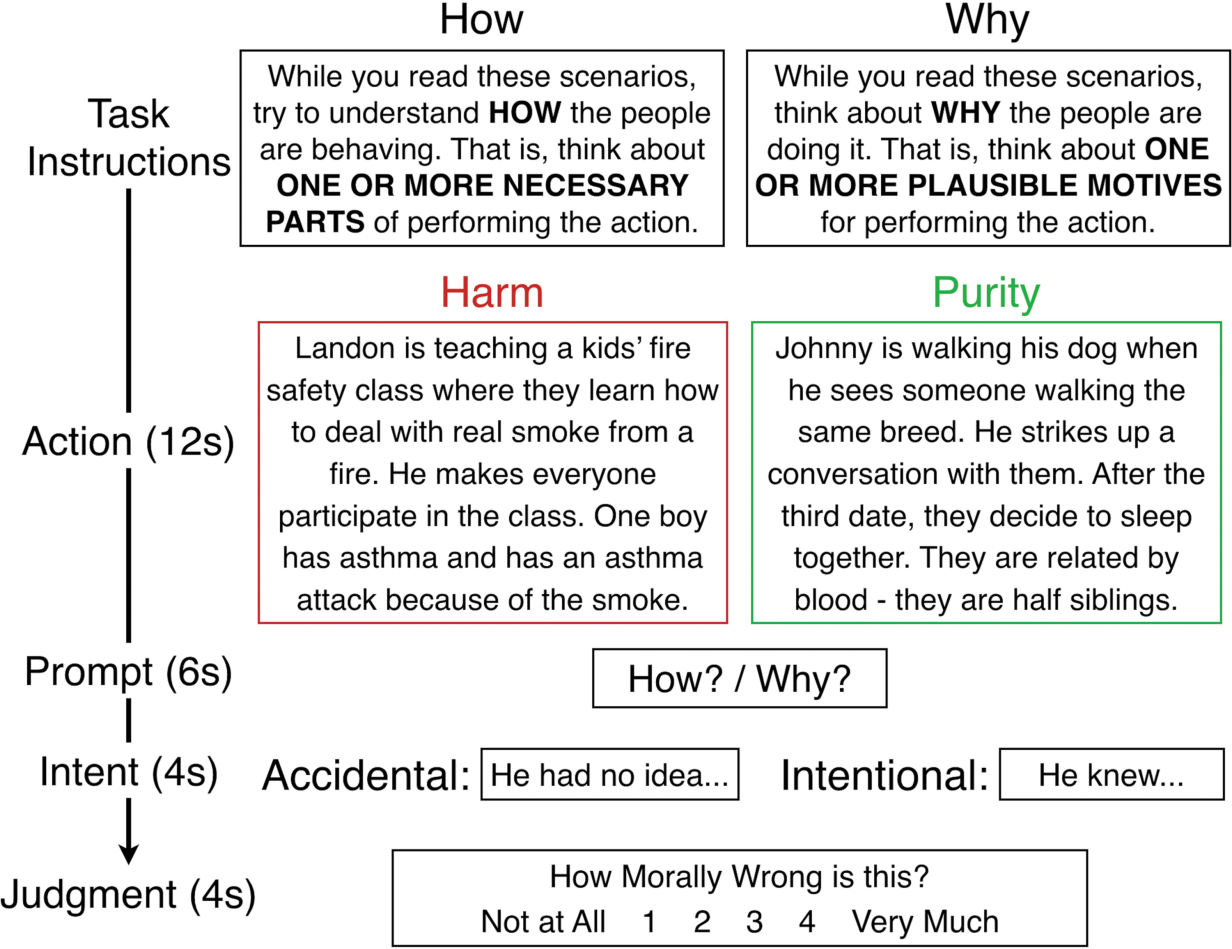
Outline of methods. Task instructions were presented at the beginning of each run. Participants saw either the accidental or intentional version of each story.

Stimuli were presented in four sequential segments, each presented alone on the screen (see Fig. 1 for a full example): action (12 s), prompt (6 s), intent (4 s) and judgment (4 s). The action section depicted the protagonist’s action. During the prompt section, participants saw a brief reminder to focus on either why or how the protagonist acted and simply pressed a button when they had a clear idea in mind. In the intent section, the action was described as being committed intentionally (e.g. the protagonist knew) or accidentally (e.g. they did not know). Finally, in the judgment section, participants used a button box to deliver their moral judgment of the protagonist’s action, on a four-point scale from 1 (not at all morally wrong) to 4 (very morally wrong).

Stimulus presentation was divided into 14 equal runs (10 stimuli per run). Each run lasted 5 min and 16 s, began and ended with 10 s of fixation and included 2–6 s of jittered fixation between each stimulus. The order of conditions was counterbalanced across runs and across participants. Word count was matched across conditions. All stimuli were presented in white font on a black background via an Apple MacBook Pro running Matlab 2012b with Psychophysics Toolbox.

Participants also completed a ToM functional localizer task (false-belief task; Dodell-Feder *et al*., 2011) consisting of 10 stories about mental states (e.g. false-belief condition) and 10 stories about physical representations (e.g. false-photograph condition; see http://saxelab.mit.edu/superloc.php for the task files). The task was presented in two 4.5 min runs, interleaved with the main experiment runs.

Following the scan session, participants completed a series of surveys. First, they were presented with scenarios from two runs they had seen in the scanner—one why run and one how run. For each scenario, they typed a short description of what they could recall thinking about when they were prompted to focus on either ‘the why’ or ‘the how’ of the protagonist’s actions. After completing that task, they completed additional surveys (Moral Foundations Questionnaire, Graham, Haidt, & Nosek, 2009; Disgust Sensitivity Scale, Haidt, McCauley, & Rozin, 1994; standard demographics survey), for exploratory purposes. These additional surveys did not correlate with any of our main results and are not discussed further here (see Supplementary Material for more discussion).

### fMRI data analyses

MRI data preprocessing and analyses were performed using SPM12 (http://www.fil.ion.ucl.ac.uk/spm) and custom software. Each participant’s data were corrected for slice timing, realigned to the first EPI, normalized to Montreal Neurological Institute brain space, spatially smoothed using a Gaussian filter (full-width half-maximum, 8 mm kernel) and high-pass filtered (128 Hz). The experimental task was modeled using a boxcar regressor convolved with a canonical hemodynamic response function. The general linear model included movement parameters as nuisance regressors.

Whole-brain and regions of interest (ROIs) analyses were conducted. A whole-brain contrast of false-belief *vs* false-photograph stories in the ToM localizer (Dodell-Feder *et al*., 2011) revealed ROIs that respond preferentially to mental states (*P* < 0.001, uncorrected, *k* > 16, value computed via 1000 iterations of a Monte Carlo simulation; Slotnick *et al*., 2003). ROIs were selected for each participant individually and defined as contiguous voxels within a 9 mm radius of the peak voxel that passed contrast threshold. Within each ROI, the average percent signal change (PSC) relative to baseline [PSC = 100 raw BOLD magnitude for (condition fixation)/raw BOLD magnitude for fixation] was calculated for each condition at each time point (averaging across all voxels in the ROI and all blocks of the same condition).

## Results and discussion

### Behavioral results

**Fig. 2:**
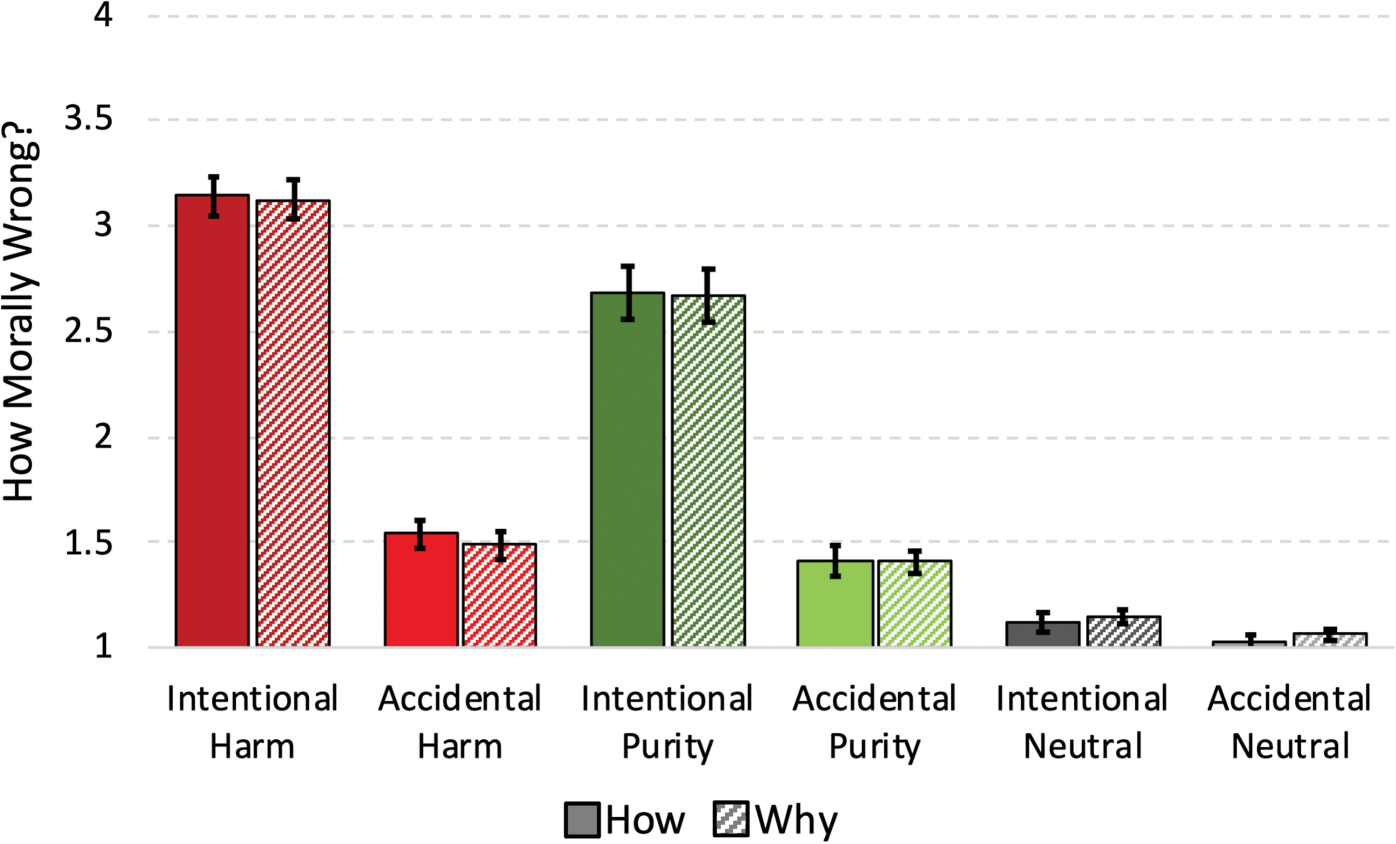
Ratings of moral wrongness made while participants were in the scanner. Ratings are broken down by intent, moral domain and task instructions. Error bars represent standard error.

Behavioral data were collected from all 29 participants. A 2 (task: why, how) × 3 (domain: harm, purity, neutral) × 2 (intent: intentional, accidental) repeated-measures analysis of variance (ANOVA) of participants’ in-scanner moral judgments revealed a main effect of domain [*F*(2,56) = 166.334, *P* < 0.001, partial η^2^ = 0.856; Fig. 2]. Harm violations were judged more harshly than purity violations, which were judged more harshly than neutral violations (harm: *M* = 2.32, s.d. = 0.34; purity: *M* = 2.04, s.d. = 0.46; neutral: *M* = 1.09, s.d. = 0.12; all *P*’s < 0.001). As expected, intentional violations were also judged more harshly than accidental violations (main effect of intent: *F*(1,28) = 321.426, *P* < 0.001, partial η^2^ = 0.920). As predicted, we also observed the key domain × intent interaction [*F*(2,56) = 147.653, *P* < 0.001, partial η^2^ = 0.841]. Consistent with prior work on the role of intentions in moral judgments of harm and purity violations (e.g. Chakroff *et al*., 2016; Young & Saxe, 2011), moral wrongness judgments reflected a smaller difference between intentional and accidental purity violations (intentional: *M* = 2.675, s.d. = 0.125; accidental: *M* = 1.411, s.d. = 0.065) than between intentional and accidental harm violations (intentional: *M* = 3.134, s.d. = 0.090; accidental: *M* = 1.514, s.d. = 0.060; both intentional and accidental neutral actions were judged close to floor: intentional: *M* = 1.131, s.d.= 0.031; accidental: *M* = 1.047, s.d. = 0.020). No other main effects or interactions were significant. Notably, this same pattern of results was found when excluding neutral scenarios (e.g. main effects of intent and domain, and domain × intent interaction, all *P*’s < 0.001). Task did not significantly change any behavioral ratings (all *P*’s > 0.35).

We also explored how participants described their thoughts in the post-scan task. Recall that participants described one why run and one how run, consisting of four harm vignettes and four purity vignettes each. Two research assistants, blind to the study’s purpose, counted the number of times participants described a vignette using mental state verbs. We analyzed the average (α = 0.88) of each research assistant’s counts of mental state verbs across conditions. A 2 (task: why, how) × 2 (domain: harm, purity) repeated-measures ANOVA of the number of mental state verbs participants used in the post-scan task revealed main effects of both task [*F*(1,28) = 23.269, *P* < 0.001, partial η^2^ = 0.454] and domain [*F*(1,28) = 15.944, *P* < 0.001, partial η^2^ = 0.363] and no interaction (*P* > 0.80). As expected, participants used more mental state verbs when describing why (*M* = 2.73, s.d. = 1.09) *vs* how (*M* = 1.51, s.d. = 1.22) a protagonist behaved. Participants also used more mental state verbs when describing harm violations (*M* = 2.31, s.d. = 1.30) than purity violations (*M* = 1.93, S.D. = 1.29).

### Functional localizer

A whole-brain analysis of scenarios describing mental states contrasted with scenarios describing physical representations replicated previous findings (Saxe and Kanwisher, 2003), revealing an increased response in four brain regions within the ToM network: RTPJ, left temporoparietal junction (LTPJ), prefrontal cortex (PC) and dorsomedial prefrontal cortex (DMPFC) (see Table 1 for peak activations using the Montreal Neurological Institute (MNI) Coordinate System). We localized these regions in the majority of participants: RTPJ (25/26 participants), LTPJ (24/26 participants), PC (25/26 participants) and DMPFC (21/26 participants).

**Table 1:** Peak MNI coordinates for ToM ROIs identified in the functional localizer

MNI coordinates						
ROI	*N* (out of 26)	*x*	*y*	*z*	No. of voxels	*t* value
RTPJ	25	52	−56	23	87	8.89
LTPJ	24	−50	−58	25	77	7.88
PC	25	1	−58	34	92	8.01
DMPFC	21	3	53	30	58	5.87

### ROI analyses

As in prior work (Chakroff *et al*., 2016), once the protagonist’s intent was revealed in the last 8 s of each experimental trial, this intent information did not modulate the average BOLD response in brain regions for ToM. The subsequent analyses therefore collapse across intentional and accidental actions. We first explored how regions within the ToM network respond to harm and purity violations across different task instructions in a 4 (ROI: RTPJ, LTPJ, PC, DMPFC) × 2 (task: why, how) × 3 (domain: harm, purity, neutral) repeated-measures ANOVA of the whole trial time course (26 s from stimulus onset to offset). We did not observe a main effect of task (*P* > 0.75); however, we did observe a main effect of ROI [*F*(3,60) = 4.438, *P* = 0.007, partial η^2^ = 0.182] as well as a main effect of domain [*F*(2,40) = 7.448, *P* = 0.002, partial η^2^ = 0.271]. Replicating prior work (Chakroff *et al*., 2016), harm violations elicited greater activity than did purity violations. We also observed a marginal ROI × task interaction [*F*(3,60) = 2.652, *P* = 0.057, partial η^2^ = 0.117], suggesting that task instructions may impact brain activity differently across regions within the ToM network.

In 2 (task: why, how) × 3 (domain: harm, purity, neutral) repeated-measures ANOVAs for each ROI individually, we observed significant main effects of domain for RTPJ [*F*(2,48) = 7.451, *P* = 0.002, partial η^2^ = 0.237], LTPJ [*F*(2,46) = 7.337, *P* = 0.002, partial η^2^ = 0.242], and a marginal effect in PC [*F*(2,48) = 2.828, *P* = 0.069, partial η^2^ = 0.105]. In each case, harm violations elicited greater activity than did both purity violations [RTPJ: *t*(24) = 3.791, *P* = 0.001; LTPJ: *t*(23) = 3.149, *P* = 0.004; PC: *t*(24) = 2.050, *P* = 0.051] and neutral actions **[**RTPJ: *t*(24) = 3.395, *P* = 0.002; LTPJ: *t*(23) = 3.806, *P* = 0.001; PC: *t*(24) = 2.077, *P* = 0.049]. Although we observed a consistent trend whereby purity violations elicited greater activity across ROIs than did neutral actions, this trend did not reach significance (all *P*’s > 0.35). We observed a main effect of task only in LTPJ [*F*(1,23) = 4.489, *P* = 0.045, partial η^2^ = 0.163], where the why task elicited greater activity than did the how task [why: *M* = 0.35, s.d. = 0.37; how: *M* = 0.07, s.d. = 0.38; *t*(23) = 2.119, *P* = 0.045]. No effects reached significance in DMPFC (*P*’s > 0.10), and the task × domain interaction did not reach significance in any ROI (all *P*’s > 0.14).

**Fig. 3:**
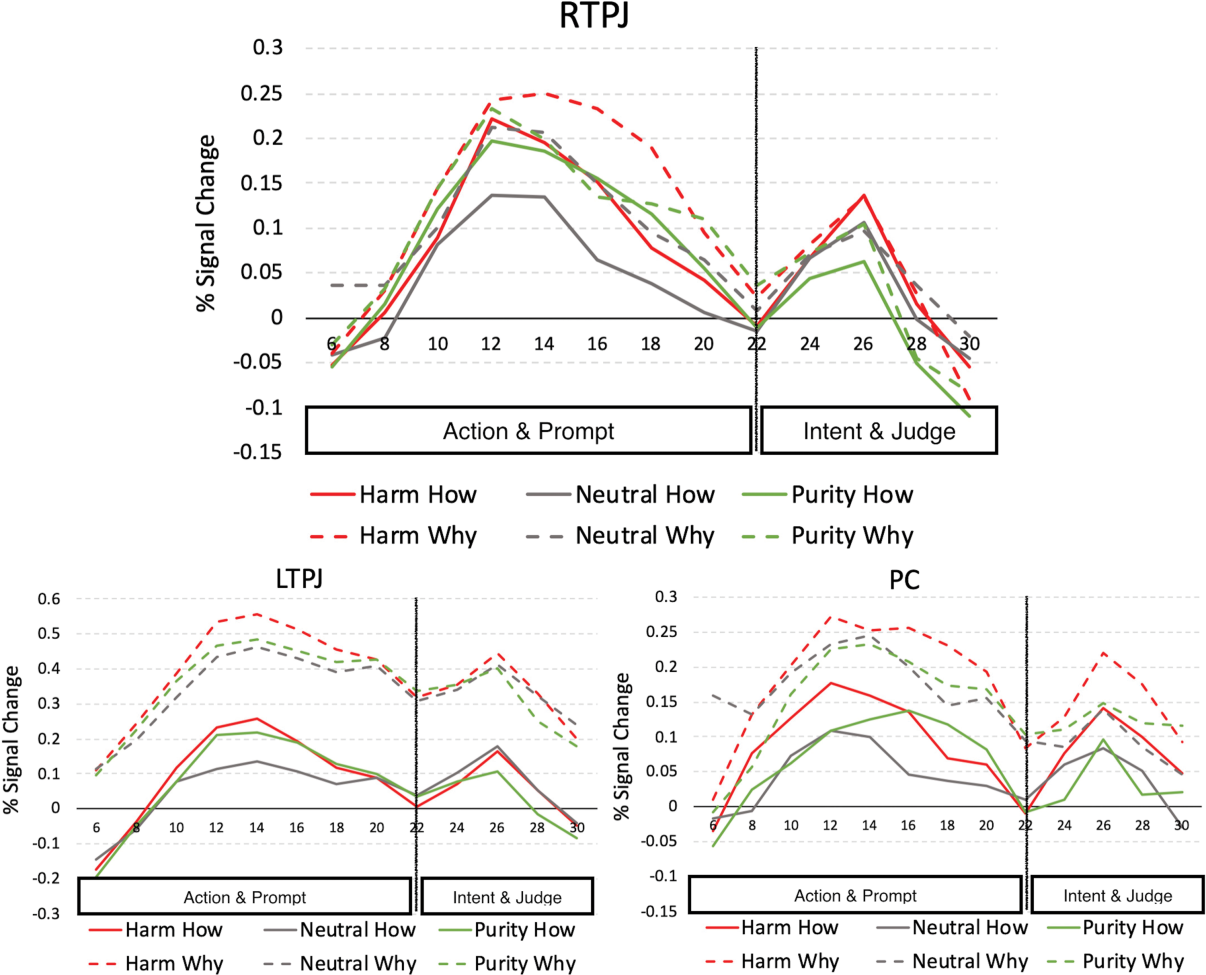
Average PSC in ROIs for each condition. The time course is marked at the boundary between part 1 (action and prompt) and part 2 (intent and judgment).

Examining the shape of the hemodynamic response across the 26 s trial length reveals two distinct peaks: one for the action and prompt segments (first 18 s) and a second for the intent and judgment segments (last 8 s; see Figure Fig. 3). Notably, prior work suggests that the difference between ToM recruitment for evaluating harm and purity violations is most robust when reading information about the violation itself compared to information about the agent’s intent (Chakroff *et al*., 2016). Given this, we next analyze the two parts of the trial separately.

In part 1 (action and prompt segments), participants read about the protagonists’ actions and considered why or how they acted. This section of the trial offers a critical test of the impact of task instructions on the processing of harm and purity violations as it is the section when we expect the largest domain difference (based on Chakroff *et al*., 2016) and when participants received the explicit prompt to attend to how or why the agent acted. A 4 (ROI: RTPJ, LTPJ, PC, DMPFC) × 2 (task: why, how) × 3 (domain: harm, purity, neutral) repeated-measures ANOVA again revealed main effects of ROI [*F*(3,60) = 4.182, *P* = 0.009, partial η^2^ = 0.173] and domain [*F*(2,40) = 9.469, *P* < 0.001, partial η^2^ = 0.321]. We also observed a significant ROI × domain interaction [*F*(6,120) = 2.843, *P* = 0.013, partial η^2^ = 0.124], as well as a marginal ROI × task interaction [*F*(2,60) = 2.691, *P* = 0.054, partial η^2^ = 0.119] and three-way ROI × domain × task interaction [*F*(6,120) = 2.142, *P* = 0.053, partial η^2^ = 0.097].

To explore these interactions further, we conducted separate 2 (task: why, how) × 3 (domain: harm, purity, neutral) repeated-measures ANOVAs for each ROI individually. We observed main effects of domain in RTPJ [*F*(2,48) = 8.147, *P* = 0.001, partial η^2^ = 0.253], LTPJ [*F*(2,46) = 10.707, *P* < 0.001, partial η^2^ = 0.318] and DMPFC [*F*(2,40) = 3.308, *P* = 0.047, partial η^2^ = 0.142]. In RTPJ, activity in part 1 did not significantly differ between harm and purity when collapsing across how and why trials (harm: *M* = 0.11, s.d. = 0.15; purity: *M* = 0.10, s.d. = 0.14; *P* > 0.30), but both elicited greater activity than did neutral actions (neutral: *M* = 0.07, s.d. = 0.15; *P*’s < 0.01). In LTPJ, activity was greater for harm than for purity [harm: *M* = 0.24, s.d. = 0.19; purity: *M* = 0.22, s.d. = 0.19; *t*(23) = 2.298, *P* = 0.031] and greater for purity than for neutral [neutral: *M* = 0.19, s.d. = 0.18; *t*(23) = 2.14, *P* = 0.043]. Finally, in DMPFC, activity was greater for harm violations than neutral actions [harm: *M* = 0.12, s.d. = 0.21; neutral: *M* = 0.09, s.d. = 0.18; *t*(20) = 2.248, *P* = 0.036]; no other comparisons reached significance (*P*’s > 0.085). We again observed a main effect of task only in LTPJ [*F*(1,23) = 4.797, *P* = 0.039, partial η^2^ = 0.173], where activity was greater in the why task (*M* = 0.366, s.d. = 0.074) than in the how task (*M* = 0.072, s.d. = 0.080). Interestingly, during part 1, we also observed a task × domain interaction in RTPJ [*F*(2,48) = 3.613, *P* = 0.035, partial η^2^ = 0.131]. Activity during the why task was relatively greater than activity during the how task for both harm violations (why: *M* = 0.13, s.d. = 0.40; how: *M* = 0.08, s.d. = 0.49) and neutral violations (why: *M* = 0.10, s.d. = 0.40; how: *M* = 0.04, s.d. = 0.49), but less so for purity violations (why: *M* = 0.11, s.d. = 0.41; how: *M* = 0.09, s.d. = 0.49; Fig. 3). Importantly, this interaction was significant when comparing harm and purity violations alone [*F*(1,24) = 4.724, *P* = 0.040, partial η^2^ = 0.164], suggesting that instructions to think about a violator’s mental states increase neural activity in a key region for ToM more for harm violations than purity violations. Thus, a difference in spontaneous mental state reasoning does not seem to explain the intent effect across harm and purity violations given that manipulating attention to an agent’s mental states appears to enhance the difference in ToM recruitment for harm *vs* purity rather than diminish it.

In part 2 (intent and judgment), information about the protagonist’s intentions was revealed and participants judged how morally wrong the protagonist’s actions were. Prior work demonstrated a smaller difference between ToM recruitment for harm and that for purity violations during this time segment than when reading about the protagonist’s actions (Chakroff *et al*., 2016); nevertheless, we explored how neural activity during this segment differed as a function of both domain and task conditions. A 4 (ROI: RTPJ, LTPJ, PC, DMPFC) × 2 (task: why, how) × 3 (domain: harm, purity, neutral) repeated-measures ANOVA again revealed main effects of ROI [*F*(3,60) = 4.388, *P* = 0.007, partial η^2^ = 0.180] and domain [*F*(2,40) = 4.290, *P* = 0.021, partial η^2^ = 0.177]. Interestingly, activity during part 2 was equally high for harm violations and neutral actions (harm: *M* = 0.12, s.d. = 0.15; neutral: *M* = 0.11, s.d. = 0.13; *P* > 0.45) and significantly lower for purity violations [*M* = 0.09, s.d. = 0.14; compared to harm, *t*(20) = 3.386, *P* = 0.003; compared to neutral, *t*(20) = 2.339, *P* = 0.030]. We also observed a significant ROI × domain interaction [*F*(6,120) = 4.815, *P* < 0.001, partial η^2^ = 0.194], a marginal ROI × task interaction [*F*(3,60) = 2.562, *P* = 0.063, partial η^2^ = 0.114] and a significant three-way ROI × domain × task interaction [*F*(6,120) = 2.392, *P* = 0.032, partial η^2^ = 0.107].

To explore these interactions further, we again conducted separate 2 (task: why, how) × 3 (domain: harm, purity, neutral) repeated-measures ANOVAs for each ROI individually. During part 2, we observed main effects of domain in RTPJ [*F*(2,48) = 6.149, *P* = 0.004, partial η^2^ = 0.204], LTPJ [*F*(2,46) = 6.390, *P* = 0.004, partial η^2^ = 0.217] and PC [*F*(2,48) = 5.157, *P* = 0.009, partial η^2^ = 0.177]. When participants were presented with the protagonist’s intent and made their moral judgments, activity was greater in PC for harm violations (*M* = 0.11, s.d. = 0.16) relative to both purity violations [*M* = 0.08, s.d. = 0.15; *t*(24) = 2.596, *P* = 0.016] and neutral actions [*M* = 0.07, s.d. = 0.14; *t*(24) = 2.459, *P* = 0.022; no difference between purity and neutral, *P* > 0.20]. In both RTPJ and LTPJ, harm violations and neutral actions elicited similarly high responses (both *P*’s > 0.70); strikingly, however, purity violations elicited less activity (RTPJ: *M* = 0.00, s.d. = 0.17; LTPJ: *M* = 0.16, s.d. = 0.19) compared to harm violations [RTPJ: *M* = 0.04, s.d. = 0.16; *t*(24) = 3.464, *P* = 0.002; LTPJ: *M* = 0.20, s.d. = 0.21; *t*(23) = 2.935, *P* = 0.007] and even neutral actions [RTPJ: *M* = 0.04, s.d. = 0.15; *t*(24) = 3.084, *P* = 0.005; LTPJ: *M* = 0.20, s.d. = 0.19; *t*(23) = 3.612, *P* = 0.001]. No main effects of task or task × domain interactions reached significance during part 2.

### Whole-brain analyses

Whole-brain random-effects analyses (voxel-wise threshold: *P* < 0.001, uncorrected; *k* > 16; cluster-wise threshold: *P* < 0.05, FWE-corrected) comparing activity during the why task *vs* the how task replicated previous work using these task manipulations (Spunt, Falk, & Lieberman, 2010; Spunt, Satpute, & Lieberman 2011). Clusters with activity that was greater during why trials (relative to how trials) were revealed within areas of the ToM network, specifically DMPFC [−12, 29, 52] and medial PC [−3, 47, 37] (Table 2). The reverse contrast revealed clusters with greater activity during how trials (relative to why trials) in premotor cortex [−24, −13, 49] and inferior parietal cortex [−54, −37, 37], regions associated with the representation and identification of actions (Mahon & Caramazza, 2009; Noppeney, 2008; Spunt, Kemmerer, & Adolphs, 2015).

**Table 2:** Regions passing threshold in whole-brain random-effects analyses (voxel-wise threshold: *P* < 0.001, uncorrected; *k* > 16; cluster-wise threshold: *P* < 0.05, FWE-corrected)

Contrast and brain region	*x*	*y*	*z*	*t*	No. of voxels
Why > how					
DMPFC (L)	−12	29	52	4.65	29
Medial PC (L)	−3	47	37	4.20	18
How > why					
Inferior parietal cortex (L)	−54	−37	37	4.71	48
Premotor area (L)	−24	−13	49	4.65	56
Harm > purity					
Primary somatosensory cortex (R)	12	−37	61	6.48	221
Middle temporal gyrus (R)	51	−22	−14	6.45	42
Rolandic operculum (L)	−45	−22	16	5.96	64
Angular gyrus (L)	−51	−55	31	4.96	57
Insula (R)	36	−22	4	4.95	184
Purity > harm					
Orbitofrontal cortex (L)	−27	32	−14	6.23	43
Inferior parietal lobule (L)	−36	−73	43	5.98	42
Cerebelum (L)	−45	−52	−26	5.91	112
Cerebelum (L)	−6	−79	−26	5.68	55
Inferior frontal gyrus, triangular part (L)	−48	38	16	5.51	57
Amygdala (L)	−27	5	−23	5.14	48
Why trials only: harm > purity					
Supplementary motor area (L)	−6	14	49	7.32	73
Postcentral gyrus (R)	12	−37	61	7.10	166
Superior temporal gyrus (L)	−42	−34	13	6.65	147
Heschl’s gyrus (R)	36	−28	7	5.47	280
Median cingulate gyrus (L)	−9	−19	40	5.41	50
Why trials only: purity > harm					
No clusters					
How trials only: harm > purity					
No clusters					
How trials only: purity > harm					
Orbitofrontal cortex (R)	24	32	−14	5.45	20
Inferior frontal gyrus, triangular part (R)	48	41	7	5.03	18
Inferior frontal gyrus, opercular part (L)	−45	8	25	4.84	26
Insula (L)	−39	−4	−14	4.78	31
Parahippocampal gyrus (L)	−18	5	−23	4.77	18
Orbitofrontal cortex (L)	−30	32	−11	4.74	28
Middle occipital gyrus (L)	−27	−61	34	4.42	21
Inferior frontal gyrus, triangular part (L)	−48	38	13	4.42	60

L, left; R, right.

We were also interested in what activity was greater when judging harm *vs* purity violations beyond the ToM network. Contrasting harm violations against purity violations revealed clusters with peak activity in primary somatosensory cortex [12, −37, 61] and regions associated with action identification (Spunt, Falk, & Lieberman, 2010). The reverse contrast (purity > harm) revealed activity in brain regions often seen during social prejudice and stereotyping, such as the amygdala [−27, 5, −23] and orbitofrontal cortex [−27, 32, −14] **(**Amodio, 2014**)**. This pattern of activity may suggest that purity violators are perceived more as outgroup members than harm violators (see Supplementary Material of Chakroff *et al*., 2016, for similar activity in a whole-brain contrast of purity against harm).

We also investigated activity for harm *vs* purity within each task separately. Within why trials only, contrasting harm against purity again revealed activity in regions associated with action identification, such as the supplementary motor area [−6, 14, 49] and superior temporal gyrus [−42, −34, 13]. No clusters passed threshold for the reverse contrast (purity against harm within why trials). Within how trials only, contrasting purity against harm again revealed activity in the amygdala [21, −18, 5] and orbitofrontal cortex [24, 32, −14]. Again, no clusters passed threshold for the reverse contrast (harm against purity within how trials).

## General discussion

Capitalizing on previous work investigating the neuroscience of action understanding (Spunt & Adolphs, 2014; Spunt, Falk, & Lieberman, 2010; Spunt, Kemmerer, & Adolphs, 2015; Spunt, Satpute, & Lieberman, 2011), we asked whether explicit instructions to pay attention to a violator’s mental states *vs* their low-level actions influenced how harm and purity violations are processed during their moral evaluation. Replicating prior work (Chakroff *et al*., 2016), harm violations elicited greater activity than did purity violations across several brain regions in the ToM network (RTPJ, LTPJ, PC). Importantly, the present study demonstrated that explicit instructions to attend to a person’s mental states did not eliminate this difference in ToM recruitment for harm *vs* purity violations. Moreover, manipulating task instructions actually *enhanced* the difference, in so far as whether or not people were explicitly instructed to attend to mental states modulated ToM activity when evaluating harm violations but not when evaluating purity violations. This was particularly true when participants first read about the actions occurring (part 1). Together, these results provide strong support that mental states play a diminished role in judgments of purity violations relative to judgments of harm violations.

The present study adds to our growing understanding of the precise role of intentions and mental state reasoning across distinct moral norms. Purity norms lie at the center of debates about whether morality can be broken down into multiple domains, each serving its own distinct function. An important first step in approaching this question is identifying whether different cognitive processes underlie purportedly distinct domains. Work in moral psychology and social neuroscience has started to provide such evidence in the context of purity norms *vs* harm norms. In particular, mental states appear to matter less in moral judgments of purity violations relative to harm violations (Barrett *et al*., 2016; Chakroff, Dungan, & Young, 2013; Dungan, Chakroff, & Young, 2017; Young & Saxe, 2011). The current results further demonstrate the robustness of this effect: while responses to harm violations change flexibly with task instructions, the same is not true for responses to purity violations. Even explicit instructions to focus on a violator’s mental states do little to change how purity violations are processed. Thus, the difference in mental state reasoning for harm *vs* purity violations does not seem to be explained by the mere presence of a salient, disgusting action that discourages people from spontaneously attending to the mental states of purity violators.

Given this evidence, the question remains of why people engage in less ToM when evaluating purity violations compared to harm violations. One possibility is that people might have difficulty understanding the mental states of a purity violator. Purity violators can be seen as weird and irrational, even relative to people that commit serious harm violations such as murder (Chakroff & Young, 2015). Given this irrationality, mental states may not be as useful for forming clear predictions about how a purity violator is likely to behave in the future, in other contexts. Notably, we see diminished activity in RTPJ for purity in part 1 of the trial when participants are first reading about the action, before they are even asked to make a moral judgment. If a purity violator’s behavior cannot be explained in terms of their underlying goals, intentions and desires, participants may not waste efforts on reasoning about a purity violator’s mental states, even when instructed to try.

Another not mutually exclusive possibility is that judgments of purity violators rely on simpler heuristics that preclude the deployment of more complex mental state reasoning. Compared to harm violations, purity violations lead to stronger person-based, dispositional attributions that do not incorporate information about mitigating circumstances (Chakroff & Young, 2015), perhaps including the violator’s innocent mental state (Barrett *et al*., 2016; Chakroff, Dungan, & Young, 2013; Dungan, Chakroff, & Young, 2017; Young & Saxe, 2011). Relatedly, perceptions of a person’s bad moral character may be more tied to purity violations than harm violations (Giner-Sorolla & Chapman, 2017; Russell & Piazza, 2015). Thus, when evaluating purity violators, participants may readily perceive them as an outgroup member. We found two pieces of evidence in line with the possibility that purity violators are seen as outgroups. First, purity violations elicited less activity in ToM brain regions than harm violations. This was true when participants were first reading about the violations as well as when they made a moral judgment. Strikingly, in this later segment when the protagonist’s intentions were revealed and participants delivered their moral judgment, purity violations elicited even less activity than did neutral actions, suggesting below-baseline ToM. This diminished activity for purity violators is consistent with the dehumanization of outgroup members (Buckels & Trapnell, 2013; Haslam, 2006), especially those who are low in warmth or elicit reactions of disgust (e.g. drug addicts, the homeless; Harris & Fiske, 2006). A second piece of evidence comes from whole-brain analyses where we see that purity violations elicit greater activity in regions associated with social prejudice and stereotyping (Amodio, 2014). This pattern further suggests that, relative to harm violators, purity violators are seen as moral outgroups.

In the current work, we were specifically interested in the role that mental state reasoning plays in moral judgments of harm *vs* purity violations. We relied on a standardized protocol for localizing brain regions involved in reasoning about a person’s beliefs and intentions (the false-belief task; Dodell-Feder *et al*., 2011). However, reasoning about people’s internal mental states involves a number of different complex processes (Carter & Huettel, 2013; Schaafsma *et al*., 2015; Schurz *et al*., 2014), and we did not include additional tasks to localize brain regions involved in other processes, such as empathy and moral disgust, that surely play a role in moral judgments of harm and purity violations. Moreover, a version of the task we used to manipulate attention to mental states while making moral judgments has also been developed as a reliable ToM localizer (the why/how task; Spunt *et al*., 2010, 2011, 2015). Instead of isolating brain regions for belief reasoning, the why/how task modulates a largely distinct left-lateralized network of brain regions involved in action explanation (Spunt & Adolphs, 2014). In a test directly comparing the two tasks in the same participants, only two regions were jointly activated by both tasks: posterior cingulate and LTPJ (Spunt & Adolphs, 2014). Interestingly, LTPJ was the only region in the current investigation in which why trials consistently evoked more activity than how trials. Importantly, attention to why *vs* how an agent behaved also increased activity in RTPJ when judging harm violations to a greater extent than when judging purity violations. Future work should further explore the contribution of other aspects of ToM (as identified by different tasks and localizers) to moral judgment.

Finally, one limitation of the current findings is their reliance on hypothetical scenarios of rare or unusual actions (i.e. harm and purity violations). While text vignettes have been a useful method of investigating moral judgments, neural activity may be markedly stronger and more widespread in real-world contexts (Camerer & Mobbs, 2017). For example, activity in anterior insula often seen in response to viewing disgusting images (Schienle *et al*., 2002; Wright *et al*., 2004) is not typically observed when reading descriptions of impure actions (Schaich Borg *et al*., 2008; Moll *et al*., 2005; Parkinson *et al*., 2011). Although we expect that our key results for the ToM network would hold for more naturalistic stimuli, future work should explore additional contributions of other networks (e.g. for empathy or disgust) to moral judgments of harm and purity violations. We also note that previous work has found that differences between judgments of harm and purity violations are not reducible to differences in perceived rarity (Chakroff & Young, 2015) or weirdness (Chakroff, Russell, Piazza, & Young, 2017) of the actions.

In sum, the current findings provide new evidence that mental states play a diminished role in judgments of purity violations relative to harm violations. This difference is robust to task instructions explicitly manipulating attention to mental states. These results constrain the potential accounts of why intentions matter less for purity violations compared to harm violations and provide further insight into the differences between distinct moral norms.

## Funding

This work was supported by a National Science Foundation Graduate Research Fellowship awarded to J.A.D. (No. 1258923) and grants awarded to L.Y. from the National Science Foundation (No. 5103831) and the John Templeton Foundation (No. 5107321).

## Supplementary Material

scan-18-403-File007_nsz048

## References

[ref1] Amodio D.M. (2014). The neuroscience of prejudice and stereotyping. Nature Reviews Neuroscience, 15(10), 670–82. 25186236 10.1038/nrn3800

[ref2] Barrett H.C., Bolyanatz A., Crittenden A.N., et al. (2016). Small-scale societies exhibit fundamental variation in the role of intentions in moral judgment. Proceedings of the National Academy of Science of the United States of America, 113(17), 4688–93. 10.1073/pnas.1522070113 PMC4855604 27035959

[ref4] Buckels E.E., Trapnell P.D. (2013). Disgust facilitates outgroup dehumanization. Group Processes & Intergroup Relations, 16(6), 771–80.

[ref5] Camerer C., Mobbs D. (2017). Differences in behavior and brain activity during hypothetical and real choices. Trends in Cognitive Sciences, 21(1), 46–56. 27979604 10.1016/j.tics.2016.11.001 PMC7769501

[ref6] Carter R.M., Huettel S.A. (2013). A nexus model of the temporal–parietal junction. Trends in Cognitive Sciences, 17(7), 328–36. 23790322 10.1016/j.tics.2013.05.007 PMC3750983

[ref7] Chakroff A., Young L. (2015). Harmful situations, impure people: an attribution asymmetry across moral domains. Cognition, 136, 30–7. 25490126 10.1016/j.cognition.2014.11.034

[ref8] Chakroff A., Dungan J., Young L. (2013). Harming ourselves and defiling others: what determines a moral domain? PloS One, 8(9), e74434. 10.1371/journal.pone.0074434 PMC3770666 24040245

[ref9] Chakroff A., Dungan J., Koster-Hale J., Brown A., Saxe R., Young L. (2016). When minds matter for moral judgment: intent information is neurally encoded for harmful but not impure acts. Social Cognitive and Affective Neuroscience, 11(3), 476–84. 26628642 10.1093/scan/nsv131 PMC4769633

[ref10] Chakroff A., Russell P.S., Piazza J., Young L. (2017). From impure to harmful: asymmetric expectations about immoral agents. Journal of Experimental Social Psychology, 69, 201–9.

[ref11] Dodell-Feder D., Koster-Hale J., Bedny M., Saxe R. (2011). fMRI item analysis in a theory of mind task. NeuroImage, 55(2), 705–12. 21182967 10.1016/j.neuroimage.2010.12.040

[ref12] Dungan J.A., Chakroff A., Young L. (2017). The relevance of moral norms in distinct relational contexts: purity versus harm norms regulate self-directed actions. PLoS One, 12(3), e0173405. 10.1371/journal.pone.0173405 PMC5344389 28278214

[ref13] Fletcher P.C., Happe F., Frith U., et al. (1995). Other minds in the brain: a functional imaging study of “theory of mind” in story comprehension. Cognition, 57(2), 109–28. 8556839 10.1016/0010-0277(95)00692-r

[ref14] Giner-Sorolla R., Chapman H.A. (2017). Beyond purity: moral disgust toward bad character. Psychological Science, 28(1), 80–91. 28078976 10.1177/0956797616673193

[ref15] Gobbini M.I., Koralek A.C., Bryan R.E., Montgomery K.J., Haxby J.V. (2007). Two takes on the social brain: a comparison of theory of mind tasks. Journal of Cognitive Neuroscience, 19(11), 1803–14. 17958483 10.1162/jocn.2007.19.11.1803

[ref16] Graham J., Haidt J., Nosek B. (2009). Liberals and conservatives use different sets of moral foundations. Journal of Personality and Social Psychology, 96, 1029–46. 19379034 10.1037/a0015141

[ref17] Haidt J., Koller S., Dias M. (1993). Affect, culture, and morality, or is it wrong to eat your dog? Journal of Personality and Social Psychology, 65, 613–28. 8229648 10.1037//0022-3514.65.4.613

[ref18] Haidt J., McCauley C., Rozin P. (1994). Individual differences in sensitivity to disgust: a scale sampling seven domains of disgust elicitors. Personality and Individual Differences, 16(5), 701–13.

[ref19] Harris L.T., Fiske S.T. (2006). Dehumanizing the lowest of the low: neuroimaging responses to extreme out-groups. Psychological Science, 17(10), 847–53. 17100784 10.1111/j.1467-9280.2006.01793.x

[ref20] Haslam N. (2006). Dehumanization: an integrative review. Personality and Social Psychology Review, 10(3), 252–64. 16859440 10.1207/s15327957pspr1003_4

[ref21] Inbar Y., Pizarro D., Knobe J., Bloom P. (2009). Disgust sensitivity predicts intuitive disapproval of gays. Emotion, 9(3), 435–9. 19485621 10.1037/a0015960

[ref22] Kestemont J., Vandekerckhove M., Ma N., Van Hoeck N., Van Overwalle F. (2012). Situation and person attributions under spontaneous and intentional instructions: an fMRI study. Social Cognitive and Affective Neuroscience, 8(5), 481–93. 22345370 10.1093/scan/nss022 PMC3682431

[ref23] Lewis G.J., Kanai R., Bates T.C., Rees G. (2012). Moral values are associated with individual differences in regional brain volume. Journal of Cognitive Neuroscience, 24(8), 1657–63. 22571458 10.1162/jocn_a_00239 PMC3383838

[ref24] Ma N., Vandekerckhove M., Baetens K., Van Overwalle F., Seurinck R., Fias W. (2011). Inconsistencies in spontaneous and intentional trait inferences. Social Cognitive and Affective Neuroscience, 7(8), 937–50. 22006990 10.1093/scan/nsr064 PMC3501697

[ref25] Mahon B.Z., Caramazza A. (2009). Concepts and categories: a cognitive neuropsychological perspective. Annual Review of Psychology, 60, 27–51. 10.1146/annurev.psych.60.110707.163532 PMC2908258 18767921

[ref26] Moll J., Oliveira-Souza R. de, Moll F.T., et al. (2005). The moral affiliations of disgust: a functional MRI study. Cognitive and Behavioral Neurology, 18(1), 68–78. 15761278 10.1097/01.wnn.0000152236.46475.a7

[ref27] Noppeney U. (2008). The neural systems of tool and action semantics: a perspective from functional imaging. Journal of Physiology, Paris, 102(1), 40–9. 10.1016/j.jphysparis.2008.03.009 18479891

[ref28] Parkinson C., Sinnott-Armstrong W., Koralus P.E., Mendelovici A., McGeer V., Wheatley T. (2011). Is morality unified? Evidence that distinct neural systems underlie moral judgments of harm, dishonesty, and disgust. Journal of Cognitive Neuroscience, 23(10), 3162–80. 21452951 10.1162/jocn_a_00017

[ref29] Piazza J., Russell P.S., Sousa P. (2013). Moral emotions and the envisaging of mitigating circumstances for wrongdoing. Cognition & Emotion, 27(4), 707–22. 23098124 10.1080/02699931.2012.736859

[ref30] Rottman J., Kelemen D., Young L. (2014). Tainting the soul: purity concerns predict moral judgments of suicide. Cognition, 130(2), 217–26. 24333538 10.1016/j.cognition.2013.11.007

[ref31] Russell P.S., Giner-Sorolla R. (2011a). Moral anger is more flexible than moral disgust. Social Psychology and Personality Science, 2(4), 360–4.

[ref32] Russell P.S., Giner-Sorolla R. (2011b). Moral anger, but not moral disgust, responds to intentionality. Emotion, 11(2), 233–40. 21500892 10.1037/a0022598

[ref48] Russell P.S., Giner-Sorolla R. (2013). Bodily moral disgust: What it is, how it is different from anger, and why it is an unreasoned emotion. Psychological bulletin, 139(2), 328. 23458436 10.1037/a0029319

[ref33] Russell P.S., Piazza J. (2015). Consenting to counter-normative sexual acts: differential effects of consent on anger and disgust as a function of transgressor or consenter. Cognition & Emotion, 29(4), 634–53. 25010550 10.1080/02699931.2014.930420

[ref34] Saxe R., Kanwisher N. (2003). People thinking about thinking people: the role of the temporo-parietal junction in “theory of mind”. NeuroImage, 19(4), 1835–42. 12948738 10.1016/s1053-8119(03)00230-1

[ref46] Saxe R., Powell L.J. (2006). It's the thought that counts: specific brain regions for one component of theory of mind. Psychological science, 17(8), 692–9. 16913952 10.1111/j.1467-9280.2006.01768.x

[ref47] Saxe R., Wexler A. (2005). Making sense of another mind: the role of the right temporo-parietal junction. Neuropsychologia, 43(10), 1891–9. 10.1016/j.neuropsychologia.2005.02.013 15936784

[ref35] Schaafsma S.M., Pfaff D.W., Spunt R.P., Adolphs R. (2015). Deconstructing and reconstructing theory of mind. Trends in Cognitive Sciences, 19(2), 65–72. 25496670 10.1016/j.tics.2014.11.007 PMC4314437

[ref3] Schaich Borg J., Lieberman D., Kiehl A. (2008). Infection, incest, and iniquity: Investigating the neural correlates of disgust and morality. Journal of Cognitive Neuroscience, 20(9), 1529–46. 18345982 10.1162/jocn.2008.20109 PMC3969035

[ref36] Schienle A., Stark R., Walter B., et al. (2002). The insula is not specifically involved in disgust processing: an fMRI study. Neuroreport, 13(16), 2023–6. 12438918 10.1097/00001756-200211150-00006

[ref37] Schurz M., Radua J., Aichhorn M., Richlan F., Perner J. (2014). Fractionating theory of mind: a meta-analysis of functional brain imaging studies. Neuroscience & Biobehavioral Reviews, 42, 9–34. 24486722 10.1016/j.neubiorev.2014.01.009

[ref38] Slotnick S.D., Moo L.R., Segal J.B., Hart J. (2003). Distinct pre- frontal cortex activity associated with item memory and source memory for visual shapes. Cognitive Brain Research, 17(1), 75–82. 12763194 10.1016/s0926-6410(03)00082-x

[ref39] Spunt R.P., Adolphs R. (2014). Validating the why/how contrast for functional MRI studies of theory of mind. NeuroImage, 99, 301–11. 24844746 10.1016/j.neuroimage.2014.05.023 PMC4111963

[ref40] Spunt R.P., Falk E.B., Lieberman M.D. (2010). Dissociable neural systems support retrieval of how and why action knowledge. Psychological Science, 21(11), 1593–8. 20959510 10.1177/0956797610386618

[ref41] Spunt R.P., Satpute A.B., Lieberman M.D. (2011). Identifying the what, why, and how of an observed action: an fMRI study of mentalizing and mechanizing during action observation. Journal of Cognitive Neuroscience, 23(1), 63–74. 20146607 10.1162/jocn.2010.21446

[ref42] Spunt R.P., Kemmerer D., Adolphs R. (2015). The neural basis of conceptualizing the same action at different levels of abstraction. Social Cognitive and Affective Neuroscience, 11(7), 1141–51. 26117505 10.1093/scan/nsv084 PMC4927039

[ref43] Van Overwalle F. (2009). Social cognition and the brain: a meta-analysis. Human Brain Mapping, 30(3), 829–58. 18381770 10.1002/hbm.20547 PMC6870808

[ref44] Wright P., He G., Shapira N.A., Goodman W.K., Liu Y. (2004). Disgust and the insula: fMRI responses to pictures of mutilation and contamination. Neuroreport, 15(15), 2347–51. 15640753 10.1097/00001756-200410250-00009

[ref45] Young L., Saxe R. (2011). When ignorance is no excuse: different roles for intent across moral domains. Cognition, 120, 202–14. 21601839 10.1016/j.cognition.2011.04.005

