# Supplementary material for: Asking ‘why?’ enhances theory of mind when evaluating harm but not purity violations
Source: Soc Cogn Affect Neurosci. 2019 Jul 3;14(7):699–708. doi: 10.1093/scan/nsz048 (PMC6778829; doi:10.1093/scan/nsz048)
Supplement: scan-18-403-File007_nsz048 [file scan-18-403-file007_nsz048.docx]

**Supplementary Material for**

**Dungan & Young, *Asking “Why?” Enhances Theory of Mind***

***When Evaluating Harm but not Purity Violations***

**1. Full Experimental Stimuli**

| **Story** | **Domain** | **Intent** | **Action** | **Intent** |
| --- | --- | --- | --- | --- |
| 1 | Harm | Accidental | Paul is about to leave his friend''s house when the carbon monoxide detector sounds. He checks the back of the detector, turns it off, and leaves the house. The detector detects dangerous levels of carbon monoxide where his friend lives. | From the back of the detector, Paul thought the detector just needed new batteries. |
| 1 | Harm | Intentional | Paul is about to leave his friend''s house when the carbon monoxide detector sounds. He checks the back of the detector, turns it off, and leaves the house. The detector detects dangerous levels of carbon monoxide where his friend lives. | From the back of the detector, Paul knew that the detector was working properly. |
| 2 | Harm | Accidental | Betty is house-sitting for a neighbor when pest control arrives. The pest control gases are very toxic, so she leaves and tells pest control to begin fumigating. The neighbor''s dog is still in the house when the fumigation begins. | From the barking sounds, Betty thought her neighbor''s dog was safely in the backyard. |
| 2 | Harm | Intentional | Betty is house-sitting for a neighbor when pest control arrives. The pest control gases are very toxic, so she leaves and tells pest control to begin fumigating. The neighbor''s dog is still in the house when the fumigation begins. | From the barking sounds, Betty knew her neighbor''s dog was still inside the house. |
| 3 | Harm | Accidental | Landon is teaching a kids'' fire safety class where they learn how to deal with real smoke from a fire. He makes everyone participate in the class. One boy has asthma and has an asthma attack because of the smoke. | Based on the boy''s health forms, Landon did not realize he had asthma. |
| 3 | Harm | Intentional | Landon is teaching a kids'' fire safety class where they learn how to deal with real smoke from a fire. He makes everyone participate in the class. One boy has asthma and has an asthma attack because of the smoke. | Based on the boy''s health forms, Landon fully realized the boy had asthma. |
| 4 | Harm | Accidental | Stella is an employee at a skydiving company. A customer wants to buy one of the store''s parachutes. Stella consults with her manager, then sells the customer the parachute. The chute failed in-house quality control and should have been tossed. | Based on what the manager said, Stella did not realize the chute was faulty. |
| 4 | Harm | Intentional | Stella is an employee at a skydiving company. A customer wants to buy one of the store''s parachutes. Stella consults with her manager, then sells the customer the parachute. The chute failed in-house quality control and should have been tossed. | Based on what the manager said, Stella definitely realized the chute was faulty. |
| 5 | Harm | Accidental | Kieran is hiking with his cousin when they find a pond. Kieran looks in his guidebook and tells his cousin it''s safe to jump in, so his cousin does. The pond actually contains dangerous bacteria and is not safe for swimming. | Because of what the guide book said, Kieran didn''t realize the pond was unsafe. |
| 5 | Harm | Intentional | Kieran is hiking with his cousin when they find a pond. Kieran looks in his guidebook and tells his cousin it''s safe to jump in, so his cousin does. The pond actually contains dangerous bacteria and is not safe for swimming. | Because of what the guide book said, Kieran definitely realized the pond was unsafe. |
| 6 | Harm | Accidental | Allison is grocery shopping for her grandmother. Spinach has been recalled for E. coli contamination, but some markets still carry it. Allison buys spinach and makes her grandmother a salad. The spinach is contaminated and makes her grandmother very sick. | Allison checked online and didn''t realize the spinach at her market might be contaminated. |
| 6 | Harm | Intentional | Allison is grocery shopping for her grandmother. Spinach has been recalled for E. coli contamination, but some markets still carry it. Allison buys spinach and makes her grandmother a salad. The spinach is contaminated and makes her grandmother very sick. | Allison checked online, so she realized the spinach at her market might be contaminated. |
| 7 | Harm | Accidental | Joseph''s classmate needs to borrow his bike. The bike just came back from a repair shop because its brakes weren''t working properly. Joseph lends it to his classmate. The brakes are still not working, and the bike is unsafe. | From his talk with the repairman, Joseph didn''t know the brakes were still broken. |
| 7 | Harm | Intentional | Joseph''s classmate needs to borrow his bike. The bike just came back from a repair shop because its brakes weren''t working properly. Joseph lends it to his classmate. The brakes are still not working, and the bike is unsafe. | From his talk with the repairman, Joseph knew that the brakes were still broken. |
| 8 | Harm | Accidental | Wendy is at a Mexican restaurant. A customer is sitting at the next table, and he starts coughing loudly. No one else is around. Wendy ignores his coughing and continues eating. He is actually choking on food and needs help. | Based on the man''s expression, Wendy didn''t realize he was choking on food. |
| 8 | Harm | Intentional | Wendy is at a Mexican restaurant. A customer is sitting at the next table, and he starts coughing loudly. No one else is around. Wendy ignores his coughing and continues eating. He is actually choking on food and needs help. | Based on the man''s expression, Wendy realized he was choking on food. |
| 9 | Harm | Accidental | Hamilton and a friend are kayaking in the ocean. It''s hot and swimming would feel great. Hamilton tells his friend to jump in while he mans the boat. There are lots of jellyfish in the water that give swimmers painful stings. | Hamilton didn''t see any jellyfish in the water at any point along their ride. |
| 9 | Harm | Intentional | Hamilton and a friend are kayaking in the ocean. It''s hot and swimming would feel great. Hamilton tells his friend to jump in while he mans the boat. There are lots of jellyfish in the water that give swimmers painful stings. | Hamilton saw there were jellyfish in the water at many points along their ride. |
| 10 | Harm | Accidental | Janice and her friend are skating in the park. She tries to rest under a tree, but a large stick is in her way. Janice tosses it aside. It lands on the path and trips her friend. He breaks his ankle. | Janice couldn''t see that her friend was skating by, so she tossed the stick. |
| 10 | Harm | Intentional | Janice and her friend are skating in the park. She tries to rest under a tree, but a large stick is in her way. Janice tosses it aside. It lands on the path and trips her friend. He breaks his ankle. | Janice saw that her friend was skating by, and she still tossed the stick. |
| 11 | Harm | Accidental | Sal is at school when a classmate asks to see what he learned in martial arts class. Sal tells him to stand back, then performs a martial arts kick. His classmate is too close and Sal kicks him in the face. | Sal could not see that his classmate was standing too close, so he kicked. |
| 11 | Harm | Intentional | Sal is at school when a classmate asks to see what he learned in martial arts class. Sal tells him to stand back, then performs a martial arts kick. His classmate is too close and Sal kicks him in the face. | Sal could see that his classmate was standing too close, but he kicked anyway. |
| 12 | Harm | Accidental | Iliana''s family is over for dinner. She wants to impress them and thinks peanuts will really improve the flavor of her dish. Iliana adds peanuts and serves everyone. Her cousin, one of her dinner guests, is severely allergic to peanuts. | Iliana didn''t know about her cousin''s allergy when she added the peanuts. |
| 12 | Harm | Intentional | Iliana''s family is over for dinner. She wants to impress them and thinks peanuts will really improve the flavor of her dish. Iliana adds peanuts and serves everyone. Her cousin, one of her dinner guests, is severely allergic to peanuts. | Iliana knew about her cousins''s peanut allergy when she added the peanuts. |
| 13 | Harm | Accidental | Olivia is climbing a tree in the park. A large wasp nest is in her way. She grabs a branch and knocks the nest out of the tree. It falls on a teenager taking a nap below the tree. | The teenager arrived after Olivia, so she didn''t see them sleeping below. |
| 13 | Harm | Intentional | Olivia is climbing a tree in the park. A large wasp nest is in her way. She grabs a branch and knocks the nest out of the tree. It falls on a teenager taking a nap below the tree. | The teenager arrived before Olivia, so she saw them sleeping down below. |
| 14 | Harm | Accidental | Ava is having family over for the 4th of July. Earlier, she found a roadside fireworks stand and bought some fireworks for her nephew to set off. When he lights one of the fireworks, it prematurely explodes, burning his hand. | Ava thought the fireworks from the stand were professionally made and would be safe. |
| 14 | Harm | Intentional | Ava is having family over for the 4th of July. Earlier, she found a roadside fireworks stand and bought some fireworks for her nephew to set off. When he lights one of the fireworks, it prematurely explodes, burning his hand. | Ava knew the fireworks were made by an amateur and could be dangerous. |
| 15 | Harm | Accidental | Liam is out with friends when their conversation lulls. To liven things up, Liam tells a joke he just heard about a boy and his puppy. His friend''s puppy was just hit by a car, and she becomes very upset. | Liam didn''t hear that his friend''s puppy died until after he told the joke. |
| 15 | Harm | Intentional | Liam is out with friends when their conversation lulls. To liven things up, Liam tells a joke he just heard about a boy and his puppy. His friend''s puppy was just hit by a car, and she becomes very upset. | Liam had just heard that his friend''s puppy died before he told the joke. |
| 16 | Harm | Accidental | Mia and a classmate are giving a formal presentation to students and faculty members. Her classmate is especially anxious about making a good impression. As they begin the presentation, an audience member points to her classmate''s open fly, mortifying him. | Mia didn''t notice his open fly and could not have warned him beforehand. |
| 16 | Harm | Intentional | Mia and a classmate are giving a formal presentation to students and faculty members. Her classmate is especially anxious about making a good impression. As they begin the presentation, an audience member points to her classmate''s open fly, mortifying him. | Mia noticed his open fly before the presentation and could have warned him. |
| 17 | Harm | Accidental | Saul''s friend is a marine, deployed in Iraq. The news lists him as a casualty in a recent attack. Saul immediately tells his friend''s parents about his death. The man who died is not their son, but has the same name. | There was no picture with the name, so Saul didn''t realize it wasn''t him. |
| 17 | Harm | Intentional | Saul''s friend is a marine, deployed in Iraq. The news lists him as a casualty in a recent attack. Saul immediately tells his friend''s parents about his death. The man who died is not their son, but has the same name. | There was a picture with the name, so Saul realized it was not him. |
| 18 | Harm | Accidental | Abigail decides to visit her sister who is babysitting. When she arrives, she can only find the baby. Abigail takes the baby home without telling her sister. When her sister cannot find the baby, she panics and calls 911. | Abigail didn''t know her sister was at the house when she took the baby. |
| 18 | Harm | Intentional | Abigail decides to visit her sister who is babysitting. When she arrives, she can only find the baby. Abigail takes the baby home without telling her sister. When her sister cannot find the baby, she panics and calls 911. | Abigail knew her sister was around and would panic if she took the baby. |
| 19 | Harm | Accidental | Maxwell is in class on the first day of school. He decides to tell a joke about an elf and a midget to introduce himself. One student in his class happens to be a dwarf and is insulted by his joke. | Maxwell didn''t see the student because he was in the back of the class. |
| 19 | Harm | Intentional | Maxwell is in class on the first day of school. He decides to tell a joke about an elf and a midget to introduce himself. One student in his class happens to be a dwarf and is insulted by his joke. | Maxwell saw the student because he was sitting right in front of him. |
| 20 | Harm | Accidental | Cynthia is in class when she notices a note on the floor next to her desk. She shows the note to the girl behind her. It is a poem written by her close friend about this girl, his secret crush. | The poem was not signed, so Cynthia really didn''t think her friend wrote it. |
| 20 | Harm | Intentional | Cynthia is in class when she notices a note on the floor next to her desk. She shows the note to the girl behind her. It is a poem written by her close friend about this girl, his secret crush. | The poem is in her friend''s writing, so Cynthia knew her friend wrote it. |
| 21 | Harm | Accidental | Lucas is taking a class on Shakespeare. On the first day, the teacher asks someone to deliver a monologue in front of the class. Lucas volunteers a classmate and the teacher makes her perform. She is terrified of public speaking. | Lucas had just met this classmate and did not realize she has this fear. |
| 21 | Harm | Intentional | Lucas is taking a class on Shakespeare. On the first day, the teacher asks someone to deliver a monologue in front of the class. Lucas volunteers a classmate and the teacher makes her perform. She is terrified of public speaking. | Lucas knew this classmate from before so he realized that she has this fear. |
| 22 | Harm | Accidental | Rena is in English class. Her teacher passes out a student''s essay to evaluate. Rena says the essay must have been written by a third-grader. The student who wrote the essay is in her class listening to her critique. | The essay was typed, so Rena completely did not realize who had written it. |
| 22 | Harm | Intentional | Rena is in English class. Her teacher passes out a student''s essay to evaluate. Rena says the essay must have been written by a third-grader. The student who wrote the essay is in her class listening to her critique. | The essay was handwritten, so Rena realized right away who had written it. |
| 23 | Harm | Accidental | Carter is ice skating with friends. One of them shows up in a frumpy sweater his grandmother gave him. Carter jokes that his grandmother has terrible taste in sweaters. His friend''s grandmother died recently and he is still very sad. | Carter had not heard anything about his friend''s grandmother recently passing away. |
| 23 | Harm | Intentional | Carter is ice skating with friends. One of them shows up in a frumpy sweater his grandmother gave him. Carter jokes that his grandmother has terrible taste in sweaters. His friend''s grandmother died recently and he is still very sad. | Carter had heard from someone that his friend''s grandmother had recently passed away. |
| 24 | Harm | Accidental | Harper receives an email from someone in her dorm saying she is planning to commit suicide, but only wants Harper to know. Harper decides she can''t bear this burden alone. She forwards the email out to everyone in her dorm. | Harper thought she was forwarding the girl''s email to the school psychiatrist only. |
| 24 | Harm | Intentional | Harper receives an email from someone in her dorm saying she is planning to commit suicide, but only wants Harper to know. Harper decides she can''t bear this burden alone. She forwards the email out to everyone in her dorm. | Harper knew that everyone in her dorm would read the girl''s private email. |
| 25 | Harm | Accidental | Dylan is in charge of his High School yearbook''s superlatives. He writes his friend in as ''least likely to succeed'' as a joke. His friend just learned that he was rejected from every college he applied to, and starts crying. | Dylan didn''t realize his friend was rejected when he wrote the superlative. |
| 25 | Harm | Intentional | Dylan is in charge of his High School yearbook''s superlatives. He writes his friend in as ''least likely to succeed'' as a joke. His friend just learned that he was rejected from every college he applied to, and starts crying. | Dylan realized that his friend was rejected when he wrote the superlative. |
| 26 | Harm | Accidental | Victoria is babysitting her young nephew. While cleaning, she finds a pile of dry pasta and construction paper and throws it out. Her nephew made it for her as a gift. He sees Victoria throw it away and starts crying. | Victoria didn''t know the pile was a gift when she threw it away. |
| 26 | Harm | Intentional | Victoria is babysitting her young nephew. While cleaning, she finds a pile of dry pasta and construction paper and throws it out. Her nephew made it for her as a gift. He sees Victoria throw it away and starts crying. | Victoria knew the pile was really a gift when she threw it away. |
| 27 | Harm | Accidental | Ryan''s neighbors are having a block party. He decides to surprise them by setting off some fireworks for the celebration. One of the party attendees is a veteran suffering from PTSD who begins to have a severe panic attack. | Ryan didn''t know setting off the fireworks would trigger the veteran''s PTSD. |
| 27 | Harm | Intentional | Ryan''s neighbors are having a block party. He decides to surprise them by setting off some fireworks for the celebration. One of the party attendees is a veteran suffering from PTSD who begins to have a severe panic attack. | Ryan knew that setting off the fireworks might trigger the verteran''s PTSD. |
| 28 | Harm | Accidental | Chloe is on a blind date. She tells him a funny story she heard about someone getting drunk and dancing around the bar in his underwear. The story is actually about Chloe''s date, who becomes incredibly embarrassed and upset. | Chloe didn''t realize the story was about her date when she brought it up. |
| 28 | Harm | Intentional | Chloe is on a blind date. She tells him a funny story she heard about someone getting drunk and dancing around the bar in his underwear. The story is actually about Chloe''s date, who becomes incredibly embarrassed and upset. | Chloe realized the story was about her date, but brought it up for fun. |
| 29 | Purity | Accidental | Johnny is walking his dog when he sees someone walking the same breed. He strikes up a conversation with them. After the third date, they decide to sleep together. They are related by blood - they are half siblings. | Johnny does not discover this until the next date after they''d already slept together. |
| 29 | Purity | Intentional | Johnny is walking his dog when he sees someone walking the same breed. He strikes up a conversation with them. After the third date, they decide to sleep together. They are related by blood - they are half siblings. | Johnny discovered this on the second date before they''d actually slept together. |
| 30 | Purity | Accidental | Maria is vacationing in Yellowstone. While hiking, she runs into someone from the same city as her. A day later, they have sex in her tent, using two forms of birth control to be safe. The person is her first cousin. | Maria didn''t realize they were cousins, since their faimiles are very distant. |
| 30 | Purity | Intentional | Maria is vacationing in Yellowstone. While hiking, she runs into someone from the same city as her. A day later, they have sex in her tent, using two forms of birth control to be safe. The person is her first cousin. | Maria realized they were cousins right away since their families are close. |
| 31 | Purity | Accidental | Dan has been chatting with someone in an online chat community every night. After several weeks, they engage in cyber sex. For this, they pleasure themselves in front of the computer''s camera. Dan''s cyber sex partner is his older sibling. | The camera only showed their body, so Dan didn''t see it was his sibling. |
| 31 | Purity | Intentional | Dan has been chatting with someone in an online chat community every night. After several weeks, they engage in cyber sex. For this, they pleasure themselves in front of the computer''s camera. Dan''s cyber sex partner is his older sibling. | The camera showed their body and face, so Dan saw it was his sibling. |
| 32 | Purity | Accidental | Jacob is at a strip club where the dancers wear masks. His co-workers buy him a private dance. The dancer is about to get undressed and Jacob is very aroused. The dancer is his own child from a former marriage. | The dancer was wearing a mask, so Jacob didn''t see it was his child. |
| 32 | Purity | Intentional | Jacob is at a strip club where the dancers wear masks. His co-workers buy him a private dance. The dancer is about to get undressed and Jacob is very aroused. The dancer is his own child from a former marriage. | The dancer took off his mask, so Jacob could see it was his child. |
| 33 | Purity | Accidental | Tyler wants to get plastic surgery and goes to a clinic his aunt suggested. The receptionist is extremely attractive and Tyler has sex with her in her office. The receptionist he has sex with is his aunt''s child, his own cousin. | Tyler''s cousin has had multiple plastic surgeries, so he did not recognize his cousin. |
| 33 | Purity | Intentional | Tyler wants to get plastic surgery and goes to a clinic his aunt suggested. The receptionist is extremely attractive and Tyler has sex with her in her office. The receptionist he has sex with is his aunt''s child, his own cousin. | Tyler''s cousin has had multiple plastic surgeries, but he still recognized his cousin. |
| 34 | Purity | Accidental | Trisha decides to reunite with her estranged brother, but when she visits, someone else answers the door. The two of them hit it off and have sex before her brother returns. This person happens to be her brother''s adult child. | Trisha thought that this person was just a house sitter, not related to her. |
| 34 | Purity | Intentional | Trisha decides to reunite with her estranged brother, but when she visits, someone else answers the door. The two of them hit it off and have sex before her brother returns. This person happens to be her brother''s adult child. | Trisha knew this person was her brother''s child, not just a house sitter. |
| 35 | Purity | Accidental | James, a 36-year old man, is in a chat room for middle-aged singles. He starts chatting with a person and their conversation gets personal very quickly. James asks if she wants to have cyber sex. She is actually a teenage girl. | James thinks she is in her early 30’s based on her profile and pictures. |
| 35 | Purity | Intentional | James, a 36-year old man, is in a chat room for middle-aged singles. He starts chatting with a person and their conversation gets personal very quickly. James asks if she wants to have cyber sex. She is actually a teenage girl. | James knows she''s a teenager and not in her 30''s before they have cybersex. |
| 36 | Purity | Accidental | Lisa is studying abroad and attends a mixer to meet foreign students. She immediately gets along with one of the boys and they later hook up. The boy is her first cousin, the son of her aunt who lives abroad. | Lisa never met her international family and didn''t know he was her cousin. |
| 36 | Purity | Intentional | Lisa is studying abroad and attends a mixer to meet foreign students. She immediately gets along with one of the boys and they later hook up. The boy is her first cousin, the son of her aunt who lives abroad. | Lisa had met her international family and knew it was her cousin. |
| 37 | Purity | Accidental | Thomas receives a strange email at work that says ''check out this awesome video!'' Thomas clicks the link, which directs to a website about extreme sexual fantasies. A graphic video of a man having sex with a horse starts playing. | Thomas had never received spam before, so he didn''t know what the link was. |
| 37 | Purity | Intentional | Thomas receives a strange email at work that says ''check out this awesome video!'' Thomas clicks the link, which directs to a website about extreme sexual fantasies. A graphic video of a man having sex with a horse starts playing. | Thomas received an email like that before, so he knew what the link was. |
| 38 | Purity | Accidental | Uric is alone in his bedroom looking through a magazine. He sees an attractive girl in an add for swimsuits. While looking at the girl, Uric pleasures himself to completion. The girl is actually his older cousin who is a model. | Uric hadn''t met his cousin, so he didn’t know it was her in the ad. |
| 38 | Purity | Intentional | Uric is alone in his bedroom looking through a magazine. He sees an attractive girl in an add for swimsuits. While looking at the girl, Uric pleasures himself to completion. The girl is actually his older cousin who is a model. | Uric had met his cousin so he knew it was her in the ad. |
| 39 | Purity | Accidental | Jaxon is on a website for people interested in BDSM and connects with a girl who shares his interests. They exchange nude pictures and talk about their mutual desires. The girl is Jaxon''s niece who is away at college. | Jaxon didn’t realize it was his niece because the photographs only showed her body. |
| 39 | Purity | Intentional | Jaxon is on a website for people interested in BDSM and connects with a girl who shares his interests. They exchange nude pictures and talk about their mutual desires. The girl is Jaxon''s niece who is away at college. | Jaxon realized it was his niece because some of the photographs showed her face. |
| 40 | Purity | Accidental | Jonathan is on Tinder and starts chatting with a girl he finds attractive. She feels the same towards him and suggests they meet. The two have dinner and end up engaging in mutual oral sex. They are actually first cousins. | Jonathan didn''t realize the girl was his cousin, because they never talked about their families. |
| 40 | Purity | Intentional | Jonathan is on Tinder and starts chatting with a girl he finds attractive. She feels the same towards him and suggests they meet. The two have dinner and end up engaging in mutual oral sex. They are actually first cousins. | Jonathan realized the girl was his cousin twhen they strated talking about their families. |
| 41 | Purity | Accidental | Pietro has been single for a while. He calls a sex hotline on the phone to get some pleasure. The woman on the other line proceeds to have phone sex with him. The woman is actually his older sister. | Pietro didn''t recognize his sister''s voice over the reception of the phone call. |
| 41 | Purity | Intentional | Pietro has been single for a while. He calls a sex hotline on the phone to get some pleasure. The woman on the other line proceeds to have phone sex with him. The woman is actually his older sister. | Pietro recognized his sister’s voice when she talked to him over the phone. |
| 42 | Purity | Accidental | Marvin is dating a woman who is epileptic, but that has not interfered with their relationship. One night, while they are having particularly intense sex, she has a seizure and dies. Marvin continues to have sex with her dead body. | Marvin didn''t realize she had died, so he continued to have sex with her. |
| 42 | Purity | Intentional | Marvin is dating a woman who is epileptic, but that has not interfered with their relationship. One night, while they are having particularly intense sex, she has a seizure and dies. Marvin continues to have sex with her dead body. | Marvin realized she had died, but he continued to have sex with her anyway. |
| 43 | Purity | Accidental | Nathan is at a friend''s party. He leaves his drink on the patio while he goes inside to mingle. Later, he gets thirsty, so he finds his drink outside and drinks it. His drink contains a fair amount of someone''s urine. | Nathan didn''t realize that someone peed in his drink, so he finished it. |
| 43 | Purity | Intentional | Nathan is at a friend''s party. He leaves his drink on the patio while he goes inside to mingle. Later, he gets thirsty, so he finds his drink outside and drinks it. His drink contains a fair amount of someone''s urine. | Nathan realized that someone peed in his drink, but he finished it anyway. |
| 44 | Purity | Accidental | Gail is replacing some flowers that neighborhood dogs dug up. She scoops up some dirt with her bare hands and gets some on her face. The dirt is actually a pile of wet steaming poop left behind by a dog. | Gail didn''t see the dog poop when she picked it up. |
| 44 | Purity | Intentional | Gail is replacing some flowers that neighborhood dogs dug up. She scoops up some dirt with her bare hands and gets some on her face. The dirt is actually a pile of wet steaming poop left behind by a dog. | Gail saw the dog poop but she picked it up anyway. |
| 45 | Purity | Accidental | Tom is at his neighbors'' house drinking tomato juice when their daughter enters the kitchen. She starts talking about vampires as Tom finishes his juice. What Tom drank was half juice, half human blood that this girl poured in. | Tom didn''t see when she poured the blood, so he drank his juice. |
| 45 | Purity | Intentional | Tom is at his neighbors'' house drinking tomato juice when their daughter enters the kitchen. She starts talking about vampires as Tom finishes his juice. What Tom drank was half juice, half human blood that this girl poured in. | Tom could see when she poured in the blood, but he drank it anyway. |
| 46 | Purity | Accidental | Christina is cooking herself rice pilaf using an old container of rice she found in her cupboard. She cooks a large cup of rice and eats it all. The rice is infested with maggots, which Christina ends up eating. | Christina didn''t see the maggots in the rice when she cooked it for dinner. |
| 46 | Purity | Intentional | Christina is cooking herself rice pilaf using an old container of rice she found in her cupboard. She cooks a large cup of rice and eats it all. The rice is infested with maggots, which Christina ends up eating. | Christina saw the maggots in the rice but cooked it for dinner anyway. |
| 47 | Purity | Accidental | Wyatt is picnicking at the beach with friends. One friend is badly sunburnt and has skin is peeling. After swimming, Wyatt eats some potato salad. While Wyatt was swimming, a breeze blew his friend''s skin flakes onto his potato salad. | Wyatt didn''t realize his food was covered in skin flakes, so he ate it. |
| 47 | Purity | Intentional | Wyatt is picnicking at the beach with friends. One friend is badly sunburnt and has skin is peeling. After swimming, Wyatt eats some potato salad. While Wyatt was swimming, a breeze blew his friend''s skin flakes onto his potato salad. | Wyatt realized his food was covered in skin flakes, but he ate it anyway. |
| 48 | Purity | Accidental | Helen is working at a free health care clinic when somebody spills coffee on her. She finds a cloth and wipes off her entire face and neck. The cloth is actually a used bandage covered in dried body fluids. | Helen didn''t think that the cloth was a used bandage, so she used it. |
| 48 | Purity | Intentional | Helen is working at a free health care clinic when somebody spills coffee on her. She finds a cloth and wipes off her entire face and neck. The cloth is actually a used bandage covered in dried body fluids. | Helen knew that the cloth was a used bandage, but she used it anyway. |
| 49 | Purity | Accidental | Will is eating at a new fast food restaurant and orders a hamburger. When it arrives he scarfs it down immediately along with fries and a soda. The burger contained the tail of a mouse that got cooked into it. | Will did not see the tiny mouse tail, so he finished his meal. |
| 49 | Purity | Intentional | Will is eating at a new fast food restaurant and orders a hamburger. When it arrives he scarfs it down immediately along with fries and a soda. The burger contained the tail of a mouse that got cooked into it. | Will saw a tiny mouse tail halfway through his meal but continued eating. |
| 50 | Purity | Accidental | A car just killed Nina''s beloved dog. To ease her mind, she cooks her housemates dinner. She chops up meat for a stew. The meat was from her dead dog. Her housemate had prepared and frozen it before it spoiled. | The meat was labeled ''beef'' so Nina didn''t realize she was eating her dog. |
| 50 | Purity | Intentional | A car just killed Nina''s beloved dog. To ease her mind, she cooks her housemates dinner. She chops up meat for a stew. The meat was from her dead dog. Her housemate had prepared and frozen it before it spoiled. | The meat was labeled ''dog'', so Nina realized that she was eating her dog. |
| 51 | Purity | Accidental | Henry is waiting for his friend to finish using the bathroom. When she finishes, Henry brushes his teeth and uses a cup on the counter to rinse his mouth. His friend just peed in that cup for her pregnancy test. | Henry thought the liquid in the cup was just mouthwash instead of urine. |
| 51 | Purity | Intentional | Henry is waiting for his friend to finish using the bathroom. When she finishes, Henry brushes his teeth and uses a cup on the counter to rinse his mouth. His friend just peed in that cup for her pregnancy test. | Henry knew that the liquid in the cup was urine from the pregnany test. |
| 52 | Purity | Accidental | Willa''s roommate recently had liposuction around her stomach. She is resting on the couch when Willa returns from the gym. Willa takes a shower and uses a bar of soap. The soap she uses is made from her roommate''s stomach fat. | The soap was labeled ''Dove'', so Willa didn''t know it was stomach fat soap. |
| 52 | Purity | Intentional | Willa''s roommate recently had liposuction around her stomach. She is resting on the couch when Willa returns from the gym. Willa takes a shower and uses a bar of soap. The soap she uses is made from her roommate''s stomach fat. | The soap was labeled ''Fat'', so Willa knew it was stomach fat soap. |
| 53 | Purity | Accidental | Alan brings soup for his sick grandpa. He opens the container and leaves to get bowls from the kitchen. Alan pours the soup and finishes his bowl. While he was in the kitchen, his grandpa coughed up some phlegm into the soup. | Alan didn''t realize that his grandpa coughed phlegm into the soup. |
| 53 | Purity | Intentional | Alan brings soup for his sick grandpa. He opens the container and leaves to get bowls from the kitchen. Alan pours the soup and finishes his bowl. While he was in the kitchen, his grandpa coughed up some phlegm into the soup. | Alan realized that his grandpa coughed phlegm into the soup. |
| 54 | Purity | Accidental | Jen is at her uncle''s house who has many strange collections. She decides to make coffee and grinds up some coffee beans from a container to brew. The container contained her uncle''s toenail clippings that she ground up with the beans. | The clippings were at the bottom, so Jen didn''t know they were in the coffee. |
| 54 | Purity | Intentional | Jen is at her uncle''s house who has many strange collections. She decides to make coffee and grinds up some coffee beans from a container to brew. The container contained her uncle''s toenail clippings that she ground up with the beans. | The clippings were at the top, so Jen knew they were in the coffee. |
| 55 | Purity | Accidental | Annette is eating powdered donuts with her cousins. One cousin has severe dandruff and has been scratching his head all day around their food. Annette takes a donut and starts licking the sugar off, eating her cousin''s dandruff as well. | Annette didn''t realize her cousin''s dandruff got on the donuts when she ate them. |
| 55 | Purity | Intentional | Annette is eating powdered donuts with her cousins. One cousin has severe dandruff and has been scratching his head all day around their food. Annette takes a donut and starts licking the sugar off, eating her cousin''s dandruff as well. | Annette realized her cousin''s dandruff got on the donuts and she ate them anyway. |
| 56 | Purity | Accidental | Simona is baking cupcakes. She gets some cream cheese from her fridge to make frosting. Her cream cheese has gone bad and is covered in spots of fuzzy mold. She covers the cupcakes in the cream cheese frosting and eats them. | The mold was white, so Simona didn''t know the cream cheese was moldy. |
| 56 | Purity | Intentional | Simona is baking cupcakes. She gets some cream cheese from her fridge to make frosting. Her cream cheese has gone bad and is covered in spots of fuzzy mold. She covers the cupcakes in the cream cheese frosting and eats them. | The mold was green, so Simona knew that the cream cheese was moldy. |
| 57 | Neutral | Accidental | Bob is walking in the park when he meets his neighbor who is walking her dog. His neighbor stops to say hi, so Bob leans down to pet her dog. Bob pets the dog and it starts wagging its tail. | Bob didn''t think his neighbor would be walking her dog that afternoon. |
| 57 | Neutral | Intentional | Bob is walking in the park when he meets his neighbor who is walking her dog. His neighbor stops to say hi, so Bob leans down to pet her dog. Bob pets the dog and it starts wagging its tail. | Bob knew that his neighbor would be walking her dog that afternoon. |
| 58 | Neutral | Accidental | Sophie is walking back home from the supermarket. She tries to step over a large puddle in middle of the sidewalk, but ends up dunking her foot in the water. Sophie is wearing rain boots, so her feet stay dry. | Sophie didn''t realize that the puddle was too big when she stepped over it. |
| 58 | Neutral | Intentional | Sophie is walking back home from the supermarket. She tries to step over a large puddle in middle of the sidewalk, but ends up dunking her foot in the water. Sophie is wearing rain boots, so her feet stay dry. | Sophie realized the puddle was too big, but she tried stepping over it anyway. |
| 59 | Neutral | Accidental | Katie is a new employee at a popular clothes store in the mall. The manager comes in and tells her to go fold and stock the new shipment of clothing. Katie had already folded the shirts before the manager asked her. | Katie didn''t know the manager would ask her to fold the new clothing. |
| 59 | Neutral | Intentional | Katie is a new employee at a popular clothes store in the mall. The manager comes in and tells her to go fold and stock the new shipment of clothing. Katie had already folded the shirts before the manager asked her. | Katie knew the manager would ask her to fold the new clothing. |
| 60 | Neutral | Accidental | Michelle is at dinner with a group of friends. As the appetizers arrive, she tells her friends about a great movie she rented last weekend. Two of Michelle''s friends already saw this movie when it came out last year. | Michelle didn''t realize that two of her friends had already seen the movie. |
| 60 | Neutral | Intentional | Michelle is at dinner with a group of friends. As the appetizers arrive, she tells her friends about a great movie she rented last weekend. Two of Michelle''s friends already saw this movie when it came out last year. | Michelle realized that two of her friends had already seen the movie. |
| 61 | Neutral | Accidental | Lily is bored and starts checking Facebook to see what her friends have been up to. She goes to the profile of an old friend she had in high school. Lily likes one of his photos he recently took in Paris. | Lily hadn''t heard that he was recently in Paris before she liked the photo. |
| 61 | Neutral | Intentional | Lily is bored and starts checking Facebook to see what her friends have been up to. She goes to the profile of an old friend she had in high school. Lily likes one of his photos he recently took in Paris. | Lily heard that he had recently been in Paris before she liked the photo. |
| 62 | Neutral | Accidental | Sam is giving a big presentation in class. He has been working on it for many weeks and feels very prepared by the time he has to present it. The teacher enjoys the presentation, especially the graphs Sam included. | Sam didn''t know the teacher would really enjoy the fancy graphs he included. |
| 62 | Neutral | Intentional | Sam is giving a big presentation in class. He has been working on it for many weeks and feels very prepared by the time he has to present it. The teacher enjoys the presentation, especially the graphs Sam included. | Sam knew the teacher would really enjoy all the fancy graphs he included. |
| 63 | Neutral | Accidental | Steven and his wife are vacationing together. This is their first vacation since their honeymoon and they finally feel relaxed and unplugged from work. Steven spends most of the week in the hotel room having sex with his wife. | Steven didn''t realize how much they needed to get away from work. |
| 63 | Neutral | Intentional | Steven and his wife are vacationing together. This is their first vacation since their honeymoon and they finally feel relaxed and unplugged from work. Steven spends most of the week in the hotel room having sex with his wife. | Steven realized just how much they needed to get away from work. |
| 64 | Neutral | Accidental | Alex goes to the phone store to replace his old phone. A very attractive woman is working the customer service desk and offers to help him. Alex signs up for a new phone contract that offers him a free phone upgrade. | Alex didn''t think he was eligible for a new phone upgrade with a contract. |
| 64 | Neutral | Intentional | Alex goes to the phone store to replace his old phone. A very attractive woman is working the customer service desk and offers to help him. Alex signs up for a new phone contract that offers him a free phone upgrade. | Alex knew that he was eligible for a new phone upgrade with a contract. |
| 65 | Neutral | Accidental | Nick has just started working at a new job. He quickly becomes friends with a girl from another department. After several months, they decide to start dating and end up sleeping together. Several years later, Nick asks her to marry him. | When they first met, Nick couldn''t tell that she would marry him one day. |
| 65 | Neutral | Intentional | Nick has just started working at a new job. He quickly becomes friends with a girl from another department. After several months, they decide to start dating and end up sleeping together. Several years later, Nick asks her to marry him. | When they first met, Nick could tell that she would marry him one day. |
| 66 | Neutral | Accidental | Stacy teaches third-grade elementary students. Today, she is working on their writing skills and has all the children write about their favorite food. One girl writes a beautiful essay about cheese and Stacey gives her paper a gold star. | Stacy didn''t realize how good of a writer this young student was. |
| 66 | Neutral | Intentional | Stacy teaches third-grade elementary students. Today, she is working on their writing skills and has all the children write about their favorite food. One girl writes a beautiful essay about cheese and Stacey gives her paper a gold star. | Stacy already realized how good of a writer this young student was. |
| 67 | Neutral | Accidental | Robert and his brother are fishing on a lake. It''s hot and the water feels great, so Robert tells his little brother to jump in for a swim. When he does, Robert''s brother sees a goose swim away from the boat. | Robert didn''t see the goose when he told his brother to swim. |
| 67 | Neutral | Intentional | Robert and his brother are fishing on a lake. It''s hot and the water feels great, so Robert tells his little brother to jump in for a swim. When he does, Robert''s brother sees a goose swim away from the boat. | Robert saw the goose when he told his brother to swim. |
| 68 | Neutral | Accidental | Phil is hungry and decides eat at his favorite fast-food restaurant for lunch. He orders two chicken sandwiches and when they arrive, he quickly eats both of them. While he eats, Phil spills some ketchup on his pants. | Phil didn''t see that some ketchup was falling from his sandwich while he ate. |
| 68 | Neutral | Intentional | Phil is hungry and decides eat at his favorite fast-food restaurant for lunch. He orders two chicken sandwiches and when they arrive, he quickly eats both of them. While he eats, Phil spills some ketchup on his pants. | Phil saw that some ketchup was falling from his sandwich, but he kept eating. |
| 69 | Neutral | Accidental | Mark is selling his old video games on craigslist. He finds an interested buyer who comes over and buys all the games he is selling. One of the games is collectible and is worth triple the price that Mark sold it for. | Mark didn''t realize that one of the games he sold was a collector''s item. |
| 69 | Neutral | Intentional | Mark is selling his old video games on craigslist. He finds an interested buyer who comes over and buys all the games he is selling. One of the games is collectible and is worth triple the price that Mark sold it for. | Mark realized that one of his games was a collector''s item, but sold it anyway. |
| 70 | Neutral | Accidental | Julia is waiting at the doctor''s office for her annual physical. Another patient enters the waiting room and sits near Julia. She is visibly sick and is coughing loudly. When the doctor calls Julia in, she cleans her hands with disinfectant. | Julia didn''t know sick people would be around while she waited for the doctor. |
| 70 | Neutral | Intentional | Julia is waiting at the doctor''s office for her annual physical. Another patient enters the waiting room and sits near Julia. She is visibly sick and is coughing loudly. When the doctor calls Julia in, she cleans her hands with disinfectant. | Julia knew she would be around sick people while she waited for the doctor. |

**2. Correlations between behavioral surveys and main results**

For transparency, we report all results for the Moral Foundations Questionnaire and Disgust Sensitivity Scale. The more participants endorsed harm as a moral value, the more harshly they tended to rate harm violations in the scanner (r(27)=.23, p=.23). This trend is stronger for judgments of intentional harms (r(27)=.25, p=.18) than judgments of accidental harms (r=.20, p=.31). The correlations between endorsement of harm and neural data were similarly weak (all r’s<.21, all *p*’s>.30). As a comparison, these effects were weaker than the correlation between endorsement of purity values and behavioral ratings of how morally wrong purity violations are (r(27)=.51, *p*=.004), though endorsement of purity values also did not correlate with any neural results (all r’s<.14, all *p*’s>.47). Disgust sensitivity was also positively correlated with hasher moral judgments of purity violations (r(27)=.59, *p*<.001)
